# CONSORT 2025 statement: 무작위 배정 임상시험 보고 가이드라인 개정판

**DOI:** 10.12771/emj.2025.00409

**Published:** 2025-07-02

**Authors:** Sally Hopewell, An-Wen Chan, Gary S. Collins, Asbjørn Hróbjartsson, David Moher, Kenneth F. Schulz, Ruth Tunn, Rakesh Aggarwal, Michael Berkwits, Jesse A. Berlin, Nita Bhandari, Nancy J. Butcher, Marion K. Campbell, Runcie C. W. Chidebe, Diana Elbourne, Andrew Farmer, Dean A. Fergusson, Robert M. Golub, Steven N. Goodman, Tammy C. Hoffmann, John P. A. Ioannidis, Brennan C. Kahan, Rachel L. Knowles, Sarah E. Lamb, Steff Lewis, Elizabeth Loder, Martin Offringa, Philippe Ravaud, Dawn P. Richards, Frank W. Rockhold, David L. Schriger, Nandi L. Siegried, Sophie Staniszewska, Rod S. Taylor, Lehana Thabane, David Torgerson, Sunita Vohra, Ian R. White, Isabelle Boutron

**Affiliations:** 1 Oxford Clinical Trials Research Unit, Centre for Statistics in Medicine, University of Oxford, Oxford, UK; 2 Department of Medicine, Women’s College Research Institute, University of Toronto, Toronto, ON, Canada; 3 United Kingdom EQUATOR Centre, Centre for Statistics in Medicine, University of Oxford, Oxford, UK; 4 Department of Clinical Research, Centre for Evidence-Based Medicine Odense and Cochrane Denmark, University of Southern Denmark, Odense, Denmark; 5 Open Patient data Explorative Network, Odense University Hospital, Odense, Denmark; 6 Centre for Journalology, Clinical Epidemiology Programme, Ottawa Hospital Research Institute, Ottawa, ON, Canada; 7 Department of Obstetrics and Gynecology, School of Medicine, University of North Carolina at Chapel Hill, Chapel Hill, NC, USA; 8 Jawaharlal Institute of Postgraduate Medical Education and Research, Puducherry, India; 9 Office of Science Dissemination, Centers for Disease Control and Prevention, Atlanta, GA, USA; 10 Department of Biostatistics and Epidemiology, School of Public Health, Center for Pharmacoepidemiology and Treatment Science, Rutgers University, New Brunswick, NJ, USA; 11 JAMA Network Open, Chicago, IL, USA; 12 Centre for Health Research and Development, Society for Applied Studies, New Delhi, India; 13 Child Health Evaluation Services, The Hospital for Sick Children Research Institute, Toronto, ON, Canada; 14 Department of Psychiatry, University of Toronto, Toronto, ON, Canada; 15 Aberdeen Centre for Evaluation, University of Aberdeen, Aberdeen, UK; 16 Project PINK BLUE-Health & Psychological Trust Centre, Abuja, Nigeria; 17 Department of Sociology and Gerontology, Miami University, Oxford, OH, USA; 18 Department of Medical Statistics, London School of Hygiene and Tropical Medicine, London, UK; 19 Nuffield Department of Primary Care Health Sciences, University of Oxford, Oxford, UK; 20 Ottawa Hospital Research Institute, Ottawa, ON, Canada; 21 Department of Medicine, Northwestern University Feinberg School of Medicine, Chicago, IL, USA; 22 Department of Epidemiology and Population Health, Stanford University, Palo Alto, CA, USA; 23 Institute for Evidence-Based Healthcare, Faculty of Health Sciences and Medicine, Bond University, Robina, QLD, Australia; 24 Departments of Medicine, of Epidemiology and Population Health, of Biomedical Data Science, and of Statistics, and Meta-Research Innovation Center at Stanford (METRICS), Stanford University, Stanford, CA, USA; 25 MRC Clinical Trials Unit at University College London, London, UK; 26 University College London, UCL Great Ormond Street Institute of Child Health, London, UK; 27 NIHR Exeter Biomedical Research Centre, Faculty of Health and Life Sciences, University of Exeter, Exeter, UK; 28 Edinburgh Clinical Trials Unit, Usher Institute-University of Edinburgh, Edinburgh, UK; 29 The BMJ, BMA House, London, UK; 30 Harvard Medical School, Boston, MA, USA; 31 Université Paris Cité, Inserm, INRAE, Centre de Recherche Epidémiologie et Statistiques, Université Paris Cité, Paris, France; 32 Clinical Trials Ontario, MaRS Centre, Toronto, ON, Canada; 33 Duke Clinical Research Institute, Duke University Medical Center, Durham, NC, USA; 34 Department of Emergency Medicine, University of California, Los Angeles, CA, USA; 35 South African Medical Research Council, Cape Town, South Africa; 36 Warwick Applied Health, Warwick Medical School, University of Warwick, Coventry, UK; 37 MRC/CSO Social and Public Health Sciences Unit & Robertson Centre for Biostatistics, Institute of Health and Wellbeing, University of Glasgow, Glasgow, UK; 38 Department of Health Research Methods Evidence and Impact, McMaster University, Hamilton, ON, Canada; 39 St. Joseph’s Healthcare Hamilton, Hamilton, ON, Canada; 40 York Trials Unit, Department of Health Sciences, University of York, York, UK; 41 Faculty of Medicine and Dentistry, University of Alberta, Edmonton, AB, Canada; 42 Université Paris Cité and Université Sorbonne Paris Nord, Inserm, INRAE, Centre for Research in Epidemiology and Statistics (CRESS), Paris, France; 43 Centre d’Epidémiologie Clinique, Hôpital Hôtel Dieu, AP-HP, Paris, France

## Abstract

**배경:**

잘 설계되고 적절하게 실행된 무작위 배정 임상시험은 의료 개입의 효과에 대한 가장 신뢰할 수 있는 증거라고 할 수 있다. 그러나 연구 보고의 질이 미흡하다는 많은 증거가 있다. CONSORT(통합 임상시험 보고 기준)는 이러한 보고의 질을 개선하기 위해 고안되었으며, 무작위 임상시험 보고서가 포함해야 할 최소한의 항목을 제시한다. CONSORT는 1996년에 처음 발표된 후 2001년과 2010년에 개정되었다. 여기에서는 최근의 방법론적 발전과 최종 사용자의 피드백을 반영하여 개정된 CONSORT 2025 statement를 소개한다.

**방법:**

문헌에 대해 범위를 검토하고 CONSORT와 관련된 경험과 이론에 바탕을 둔 프로젝트별 데이터베이스를 개발하여 점검표의 잠재적 변경 목록을 생성하였다. 이 목록은 기존 CONSORT 확장판(유해성, 결과, 비약물적 치료)의 주 저자들과, 관련 보고 지침들(TIDieR), 그리고 개인적인 의견 교환을 포함한 기타 출처에서 제공된 권고사항으로 보강되었다. 점검표의 잠재적 변경 목록은 317명이 참여한 대규모 국제 온라인 3 라운드 델파이 설문조사를 통해 평가되었으며, 초청된 30명의 국제 전문가가 참여한 이틀 간의 온라인 전문가 합의 회의에서 논의되었다.

**결과:**

CONSORT 점검표를 대폭 변경하였다. 새로운 항목 7개를 추가하고, 3개 항목을 수정하고, 1개 항목은 삭제했으며, 주요 CONSORT 확장판의 여러 항목을 통합하였다. 또한 오픈 사이언스에 대한 새로운 섹션을 추가하여 CONSORT 점검표를 재구성하였다. CONSORT 2025 statement는 무작위 임상시험 결과를 보고할 때 반드시 포함해야 하는 30개 항목의 점검표와 임상시험 참여자의 흐름을 문서화하기 위한 다이어그램으로 구성되어 있다. 또한 각 항목의 핵심 요소를 도출하여 글머리 기호 형식으로 정리한 확장형 점검표도 개발하여 CONSORT 2025를 용이하게 이행할 수 있도록 하였다.

**결론:**

저자, 편집인, 심사자 및 기타 잠재적 사용자는 무작위 임상시험의 원고를 작성하고 평가할 때 CONSORT 2025를 사용하여 임상시험 보고서를 명확하고 투명하게 작성하도록 해야 한다.

## 저자 요약

- 무작위 임상시험을 정확하게 해석할 수 있도록 독자에게 임상시험의 방법과 결과에 대한 완전하고 투명한 정보를 제공해야 한다.

- CONSORT 2025 statement는 방법론적 발전과 최종 사용자의 피드백을 반영하여 무작위 배정 임상시험 결과 보고에 대한 개정된 지침을 제공한다.

- CONSORT 2025 statement는 30개 항목으로 구성된 필수 항목 점검표, 임상시험 참여자의 흐름을 문서화하기 위한 다이어그램, 각 점검표 항목의 핵심 요소를 자세히 설명하는 확장된 점검표로 구성되어 있다.

- 저자, 편집인, 검토자 및 기타 잠재적 사용자는 무작위 배정 임상시험 원고를 작성하고 평가할 때 CONSORT 2025를 사용하여 임상시험 보고서가 명확하고 투명하게 작성되도록 해야 한다.

## 서론

“독자가 연구과정에 무슨 일이 있었는지 유추해야 할 필요가 없도록 명확하게 알려야 한다.” Douglas G. Altman [1]

무작위 배정 임상시험은 적절하게 설계, 수행, 분석, 보고되었을 때 일반적으로 의료 중재를 평가하는 데 있어 가장 높은 수준의 근거로 간주된다. 무작위 배정 임상시험의 질에 대한 비판적 평가는 임상시험의 설계, 수행, 분석 및 결과를 철저하고 정확하게 보고할 때만 가능하다. 독자가 임상시험을 정확하게 해석하려면 임상시험의 방법과 결과에 대한 완전하고 투명한 정보가 필요하다. 그러나 무작위 배정 임상시험 보고의 완전성이 불충분하며[2,3] 이러한 불완전한 보고는 중재효과의 추정치에 편향을 초래할 수 있다는 광범위한 증거가 있다[4]. 마찬가지로, 명확하고 투명한 임상시험 프로토콜을 갖추는 것은 중요하다. 일차 결과와 같이 임상시험에 사용될 방법을 미리 지정함으로써, 미공개 사후 변경이 이루어질 가능성을 줄이기 때문이다[5].

무작위 임상시험 보고를 개선하려는 노력은 1990년대 초에 탄력을 받아, 1994년 표준화된 임상시험 보고(Standardised Reporting of Trials, SORT) 및 아실로마르 이니셔티브(Asilomar initiatives)가 탄생하였다. 이러한 이니셔티브는 1996년 CONSORT(임상시험 보고 통합 기준) statement 발표로 이어졌고[6], 2001년에는 설명 및 상세 문서와 함께 개정되었다[7,8]. 이후 2010년에 CONSORT가 개정되었고[9], 이에 대한 설명 및 상세 문서도 함께 개정되었다[10]. 마찬가지로 임상시험 프로토콜에서 완전하고 투명한 보고가 부족하다는 문제로 인해 SPIRIT(표준 프로토콜 항목: 중재적 임상시험을 위한 권고사항) statement가 개발되어 2013년에 발표되었으며[11], 이 statement의 기본 원칙에 대한 설명 및 상세 문서도 함께 발표되었다[12].

CONSORT는 전 세계 수많은 학술지와 세계의학편집인협회(Word Association of Medical Editors), 국제의학학술지편집인위원회(International Committee of Medical Journal Editors), 미국 과학학술지편집인협의회(Council of Scientific Editors) 등 저명한 편집 관련 단체들의 지지를 받고 있다. 학술지 내 CONSORT 도입은 무작위 배정 임상시험 보고의 질 향상과 관련이 있는 것으로 나타났다. 일부 근거에 따르면, 학술지가 CONSORT를 지지하는 경우 보고의 질이 향상되며, 시간이 지남에 따라 보고가 더욱 개선되는 것으로 나타났다[2,13-15]. 16,604건의 임상시험에 대한 50건의 평가를 종합한 코크란 리뷰(Cochrane review)에서 학술지의 CONSORT 지지 여부와 학술지가 발표한 임상시험 보고 사이의 연관성을 평가했는데, 27개의 CONSORT 점검표 항목 중 25개 항목이 CONSORT를 지지하는 학술지에 발표된 임상시험에서 지지하지 않는 학술지보다 더 완전하게 보고되었다[2,14]. 그러나 인과 관계를 증명할 수는 없다. 최소한 CONSORT는 많은 최종 사용자(예: 저자, 학술지 편집인, 동료 심사자)에게 무작위 배정 임상시험에서 신중하고 철저한 보고가 얼마나 중요한지에 대한 인식을 일깨워주었다.

SPIRIT와 CONSORT는 각각 완료된 무작위 임상시험의 프로토콜과 1차 보고서에 포함되어야 하는 필수 항목의 점검표와, 임상시험 참여자의 흐름을 문서화한 도표로 구성된, 근거 기반 가이드라인이다. 이 지침은 임상시험 계획서와 보고서가 명확하고 투명하게 작성될 수 있도록, 임상시험 보고가 포함해야 하는 최소한의 정보에 대한 지침을 작성자에게 제공한다. 또한 각 점검표 항목의 의미와 근거, 좋은 보고의 예, 그리고 가능하다면 관련된 경험적 근거까지 제공하는 설명서 및 상세 문서를 함께 발행한다.

2020년 1월, 영국 옥스퍼드에서 SPIRIT과 CONSORT의 임원진이 모였다. 이들은 SPIRIT와 CONSORT statements는 개념적으로 연결되어 있고, 내용이 중복되며, 전파 및 이행 전략도 유사하기 때문에 두 그룹이 함께 일하는 것이 더 효과적이라고 판단하여 하나의 그룹을 구성하였다. CONSORT 2025 statement는 *The BMJ, JAMA, The Lancet, Nature Medicine, PLoS Medicine*에 동시에 게재될 예정이다.

## SPIRIT 및 CONSORT statement 개정 결정

SPIRIT와 CONSORT는 생동하는(living) 가이드라인으로, 새로운 근거, 방법론적 발전, 사용자의 피드백을 반영하여 주기적으로 개정하는 것이 매우 중요하다. 그렇지 않으면, 시간이 지남에 따라 그 가치와 유용성이 감소할 수 있다[16]. SPIRIT 2013과 CONSORT 2010 statement를 함께 개정하는 것은 두 점검표의 일관성을 더욱 높이고, 임상시험의 설계, 수행, 분석, 결과 등 임상시험 계획서부터 최종 출판에 이르기까지의 과정에서 사용자에게 일관된 지침을 제공할 수 있는 기회이기도 하다. 보고 과정을 조화롭게 만드는 것은 사용성과 준수도를 향상하고, 더 완전한 보고로 이어질 것이다[17]. 여기에서는 개정된 CONSORT 2025 statement를 소개하며, 개정된 SPIRIT 2025 statement는 별도로 발표되었다[18].

## CONSORT 2025의 개발

CONSORT statement 개정에 사용된 방법은 보건 연구 지침 개발자를 위한 EQUATOR 네트워크 지침을 따랐으며[19], 이는 다른 곳에서 자세히 설명하였다[20,21]. 간단히 설명하자면, 먼저 문헌에 대한 범위 검토를 수행하여 수정 및 추가를 제안하거나 CONSORT 2010의 강점과 과제를 성찰하는 기존 출판 의견을 확인했으며, 그 결과는 별도로 발표되었다[22]. 또한 CONSORT 및 무작위 배정 임상시험의 편향 위험과 관련된 경험적, 이론적 증거를 위한 프로젝트별 데이터베이스(SCEBdb)를 개발하였다[23]. 범위 검토에서 확인된 근거는, 점검표 항목이 모든 임상시험(유해성[24], 결과[25]) 또는 상당수의 임상시험[26](비약물적 치료[27])에 적용되는 특정 주요 기존 CONSORT 확장(extension)의 주요 저자가 제공한 권고사항 및 근거와 결합되었으며, 기타 관련 보고 가이드라인(중재 설명 및 복제를 위한 템플릿[template for intervention description and replication, TIDieR] [28]) 및 기타 출처(예: 개인 커뮤니케이션)의 권고와도 결합되었다.

기존 CONSORT 2010 점검표를 출발점으로 삼고, 범위 검토 및 권고사항에서 수집한 근거를 사용하여 점검표에 대한 잠재적 수정 또는 추가 목록을 작성하였다. 이 잠재적 변경사항 목록을 최종 사용자에게 제시하고, 1라운드에 317명, 2라운드에 303명, 3라운드에 290명이 응답한 대규모 국제 온라인 델파이 설문조사에서 피드백을 받았다. 델파이 참가자는 기존의 SPIRIT 및 CONSORT 협력체와 전문 연구 네트워크 및 학회를 통해 선정하였다. 또한 SPIRIT-CONSORT 개정 프로젝트 웹사이트에 관심 표명 양식을 마련하여 참가자를 모집하였다. 최종 사용자의 역할은 다양했는데, 통계학자/방법론자/역학자(n=198), 체계적 문헌고찰자/가이드라인 개발자(n=73), 임상시험 조사자(n=73), 임상의(n=58), 학술지 편집인(n=47), 환자 대표(n=17)가 가장 많았다(복수 소속 가능). 세 차례에 걸친 델파이 설문조사에서, 참가자들에게 개정된 CONSORT 점검표에 포함된 각 항목에 어느 정도 동의하는지 5점 리커트 척도로 평가하도록 요청하였다. 각 항목에 대한 의견을 제시하고 새로운 점검표 항목을 추가로 제안할 수 있는 무료 텍스트 상자도 제공하였다.

델파이 설문조사 결과는 2023년 3월 1일과 2일 이틀간 Zoom (Zoom Video Communications Inc.)을 통해 진행된 온라인 전문가 합의 회의에서 발표되고 논의되었는데, 여기에는 델파이 설문조사에 포함된 다양한 이해관계자 그룹을 대표하는 초청 국제 참가자 30명이 참석하였다. 이 회의에서는 새로 추가되거나 수정된 CONSORT 점검표 항목에 대해 논의하고 합의를 모색하였다. 의견 차이가 있는 항목에 대해서는 Zoom을 통한 익명 투표로 지지 수준을 파악하였다. 이러한 투표는 단지 권고적 성격으로, 공식적인 합의 임계값(threshold)은 지정되지 않았다.

전문가 합의 회의 이후, 실무진은 2023년 4월 25일과 26일 이틀간 옥스퍼드에서 대면 서면 회의를 개최하여 새롭게 추가되거나 수정된 CONSORT 점검표 각 항목의 형식과 문구를 검토하고 합의하였다. 그런 다음 점검표 초안을 컨센서스 회의 참가자들이 회람하고, 각 항목이 그룹 합의를 반영하는지 또는 추가 설명이 필요한지를 확인하였다. 이 피드백을 바탕으로 임원진에서 CONSORT 항목을 추가로 수정하였다. 최종 수정된 항목은 임상시험 보고서에 포함해야 할 최소한의 내용을 다루고 있으나, 이는 향후 저자들이 중요하다고 생각하거나 복제를 용이하게 하는 추가 정보를 포함하지 못하게 하는 것은 아니다. 집행 그룹의 구성원과 30명의 초청된 합의 회의 참가자는 본 원고의 저자이며, 이들의 이름은 원고 말미에 기재되어 있다.

## CONSORT 2025의 주요 변경사항

CONSORT 2025 점검표에 여러 가지 실질적인 변경사항을 적용하였다(박스 1 참조). 7개의 새로운 점검표 항목을 추가하고, 3개 항목을 수정했으며, 1개 항목을 삭제했다. 아울러 주요 CONSORT 확장(유해성[24], 결과[25], 비약물학적 치료[27]) 및 기타 관련 보고 가이드라인(TIDieR [28])의 여러 항목을 통합하였다. 임상시험 등록(항목 2), 임상시험 프로토콜 및 통계 분석계획에 접근할 수 있는 위치(항목 3), 비식별화된 참가자 수준의 데이터 공유(항목 4), 자금 및 이해관계(항목 5) 등 개념적으로 연결된 항목을 포함하는 오픈 사이언스에 대한 새로운 섹션을 추가하여 CONSORT 점검표도 재구성하였다. 또한 CONSORT와 SPIRIT 점검표 항목 간의 문구를 통일하고, 일부 항목의 문구를 명확하고 간결하게 다듬었다. CONSORT 2010 점검표와 CONSORT 2025 점검표의 변경사항을 자세히 비교하려면 Supplement 1을 참조하면 된다. 더불어 CONSORT 설명 및 상세화 문서를 개정하여[29], 각 CONSORT 2025 점검표 항목에 대한 근거와 과학적 배경을 설명하고 우수 보고 사례를 게시하였다.

**박스 1. CONSORT 2025의 주요 변경사항 요약**

**새로운 점검표 항목 추가**

- 항목 4: 비식별화된 개별 참여자 데이터, 통계 코드 및 기타 자료에 접근할 수 있는 위치와 방법을 포함한 데이터 공유에 관한 항목 추가

- 항목 5b: 원고 작성자의 재정적 및 기타 이해상충에 관한 항목 추가

- 항목 8: 임상시험의 설계, 수행 및/또는 보고에 환자 및/또는 대중이 어떻게 참여했는지에 대한 항목 추가

- 항목 12b: 해당되는 경우, 시험기관 및 중재를 제공하는 개인의 자격 기준에 대한 항목 추가

- 항목 15: 위해 및 기타 의도하지 않은 영향 평가방법에 대한 항목 추가

- 항목 21: 각 분석에 포함되는 대상(예: 모든 무작위 참가자) 및 그룹(항목 21b), 분석에서 누락된 데이터를 처리하는 방법(항목 21c)을 정의하는 항목 추가

- 항목 24: 중재와 비교군이 실제로 어떻게 시행되었는지에 대한 내용(항목 24a), 임상시험 기간 동안 받은 동반 치료의 세부 사항(항목 24b) 등 중재 제공에 관한 항목 추가

**전면 수정된 점검표 항목**

- 항목 3: 임상시험 계획서 외에 통계 분석계획에 접근할 수 있는 위치를 포함하도록 항목 수정

- 항목 10: 임상시험 시작 후 미리 지정되지 않은 결과 또는 분석을 포함한 임상시험의 중요한 변경사항을 보고에 포함하도록 항목 수정

- 항목 26: 1차 및 2차 각 결과별로 분석에 포함된 참가자 수와, 각 시점에 사용 가능한 데이터를 가진 각 치료군별 참가자 수를 명시하도록 항목 수정

**점검표 항목 삭제**

- 임상시험 결과의 일반화 가능성에 대한 항목 삭제(임상시험 제한사항[30번 항목]에 통합)

**주요 CONSORT 확장의 점검표 항목 통합**

- 위해[24]를 평가 및 분석한 방법(항목 7, 15, 21a, 23a, 27), 결과[25]를 측정 및 분석한 방법(항목 14, 26), 중재[27,28]와 비교군이 실제로 어떻게 시행되고 누가 시행했는지(항목 24) 보고와 관련된 항목 추가

**점검표 항목의 구조 및 구성**

- 임상시험 등록(항목 2), 임상시험 프로토콜 및 통계 분석계획에 접근할 수 있는 위치(항목 3), 비식별화된 참가자 수준의 데이터 공유(항목 4), 자금 및 이해상충(항목 5) 등 개념적으로 연결된 항목을 포함한 오픈 사이언스에 대한 섹션을 신설하여 점검표를 재구성

- 일부 CONSORT 점검표 항목의 문구를 SPIRIT 점검표 항목의 문구와 통일하고, 그 반대도 적용

- 일부 항목의 문구를 명확하고 간결하게 수정

또한 CONSORT 2025의 원활한 이행을 돕기 위해, 각 항목의 핵심 요소를 도출하여 글머리 기호 형식으로 정리한 확장 버전 CONSORT 2025 점검표를 개발하였다. 이는 COBWEB (CONSORT 기반 웹 도구) [30] 및 COBPeer (CONSORT 기반 동료 검토 도구) [31] 연구에서 제안하고 체계적 문헌고찰 보고를 위한 2020 PRISMA 지침[32]에서 사용한 모델과 유사하다. 확장된 점검표는 CONSORT 2025 설명 및 상세화 문서에 제시된 요소를 요약한 버전으로[29], 예시와 참조는 제외되었다(Supplement 2).

## CONSORT 2025의 범위

CONSORT 2025 statement는 30개 항목의 점검표로 구성되어 있으며, 무작위 배정 임상시험 보고서에 포함되어야 할 최소한의 항목(표 1)과 임상시험 참여자의 흐름을 문서화하는 도표를 제공한다(그림 1). CONSORT 2025 statement는 CONSORT 2025 설명 및 상세 문서와 함께 사용할 것을 강력히 권장한다[29]. CONSORT 2025 statement는 CONSORT 2010 statement를 대체하므로, CONSORT 2010 statement는 더 이상 사용되어서는 안 된다. 학술지 편집인과 출판사는 저자 지침을 개정하여 CONSORT 2025를 참조하도록 해야 한다. CONSORT 2025는 모든 무작위 배정 임상시험 보고에 대한 지침을 제공하지만, 가장 일반적인 유형인 두 집단 병렬 설계(two-group parallel design)에 중점을 둔다(그림 1).

CONSORT의 확장판은 다양한 유형의 임상시험 설계, 데이터 및 중재유형의 보고와 관련된 방법론적 문제를 해결하기 위해 개발되었다. 임상시험 설계 확장에는 적응 설계[33], 군집 시험[34], 교차 시험[35], 초기 단계 시험[36], 요인 시험[37], 비열등성 및 동등성 시험[38], 실용적 시험[39], 다군 시험[40], n-of-1 시험[41], 시범 및 타당성 시험[42], 사람 내 무작위 배정 시험[43]에 대한 권장사항이 포함된다. 그 외 비약물학적 치료[27], 결과[25], 환자 보고 결과[44], 대리 결과[45], 사회적 및 심리적 개입[46], 피해[24], 초록[47], 건강 형평성[48] 등이 포함된다. 우리는 이러한 확장판의 책임자들과 협력하여, 개정된 CONSORT 2025 statement에 부합하는 프로세스를 구현할 것이다. 독자들은 당분간 기존 버전의 관련 CONSORT 확장판을 사용할 것을 권장한다.

## 시사점 및 한계

CONSORT 2025 statement의 목적은 명확하고 완전하며 투명한 방식으로 임상시험을 보고하기 위해 저자가 포함해야 하는 내용에 대한 최소한의 권장사항을 제공하는 것이다[9,10]. 독자, 동료 심사자, 임상의, 가이드라인 작성자, 환자 및 일반인, 그리고 편집인 또한 CONSORT 2025를 활용하여 무작위 배정 임상시험의 보고를 평가하는 데 도움을 받을 수 있다. 또한 원고 제출과정에서, 각 점검표 항목이 원고의 어디에 보고되어 있는지를 명시한 완성된 CONSORT 2025 점검표를 제출하고, 이를 보충자료의 일부로 업로드할 것을 강력히 권장한다[49]. 모호함이나 누락 없이 수행한 작업과 발견한 내용을 명시적으로 설명하는 것이 모든 독자의 이익에 가장 부합한다[9].

CONSORT 2025와 SPIRIT 2025에는 임상시험 설계, 수행 또는 분석에 대한 권장사항을 포함하고 있지 않지만, 그럼에도 불구하고 여기에 포함된 권장사항은 고려해야 할 주요 이슈를 강조함으로써 연구자들이 임상시험을 설계, 수행 및 분석하는 데 도움을 줄 수 있다. 그리고 SPIRIT 및 CONSORT statement를 함께 개정하는 것은 두 점검표의 보고 내용을 일치시키고 임상시험 계획서부터 최종 출판까지 임상시험 설계, 수행 및 분석 보고에 대한 일관된 지침을 사용자에게 제공할 수 있는 기회이기도 했다[17]. 따라서 임상시험 계획서의 명확하고 투명한 보고는 결과적으로 적절하게 설계되고 잘 수행된 임상시험을 촉진하는 데 도움이 될 것이다. 또한 임상시험 결과를 투명하게 보고하면, 연구 결함이 존재하는 경우 이를 밝혀내고 그 유병률과 심각성을 더 잘 추정할 수 있다. 그러나 중요한 것은 CONSORT 2025가 품질 평가도구로 사용되지는 않는다는 점이다. CONSORT 2025의 내용은 오히려 무작위 배정 임상시험의 내부 및 외부 유효성과 관련된 보고 항목에 초점을 맞추고 있다.

CONSORT 2025에서는 무작위 임상시험 보고를 위한 엄격한 구조를 제안하지 않는다. 대신, 논문의 형식은 학술지의 개별 스타일과 “저자를 위한 지침”을 준수해야 한다. 저자는 논문의 어딘가에 점검표 항목을 충분히 상세하고 명확하게 언급해야 한다[9]. 또한 임상시험 방법과 결과를 일부 인쇄 학술지 논문의 일반적인 분량 한도보다 더 자세히 보고할 수 있도록, 추가적인 온라인 보충자료의 사용을 권장한다. 전체 데이터 및 코드 공유는 또 다른, 더 높은 수준의 투명성을 제공하며, 무작위 임상시험에서 이러한 일이 발생했는지 또는 발생할 계획인지(예: 일정 시간 후)에 대한 자세한 정보를 제공할 것을 권장한다.

CONSORT는 실제 임상시험 설계와 수행, 분석을 반영하는 명확하고 투명한 보고를 촉구한다. 고품질의 보고는 재현성과 관련된 문제를 고려할 때 중요한 단계이다[50]. 임상시험 저자는 수행된 사항을 자세히 설명하고, 수행되지 않았거나 수정된 사항이 있는 경우, 이를 인정하여 임상시험 계획서, 통계 분석 계획서 및 임상시험 등록부에 보고된 정보와 일치하도록 하는 것이 좋다. 연구자, 연구 수련생, 학술지 편집인, 동료 심사자를 대상으로 하는 추가 리소스 및 교육 자료를 포함하여 CONSORT 및 SPIRIT statement에 대한 자세한 정보를 제공하기 위해 SPIRIT-CONSORT 공동 웹사이트(https://www.consort-spirit.org/)가 개설되었다. 이 웹사이트에는 환자와 대중을 대상으로 한 자료도 포함되어 있어, 무작위 배정 임상시험의 명확하고 투명한 보고의 중요성과 근거 기반 의료 제공에서의 중요성을 설명한다.

CONSORT 2025는 새로운 증거와 새로운 관점을 반영하기 위해 주기적으로 개정되는, 생동하는(living) 가이드라인이다. 이러한 접근방식은 저자, 환자 및 일반인, 학술지 편집인, 동료 심사자 등 최종 사용자에게 지침의 관련성을 유지하는 데 중요하다.

## Figures and Tables

**그림 1.**
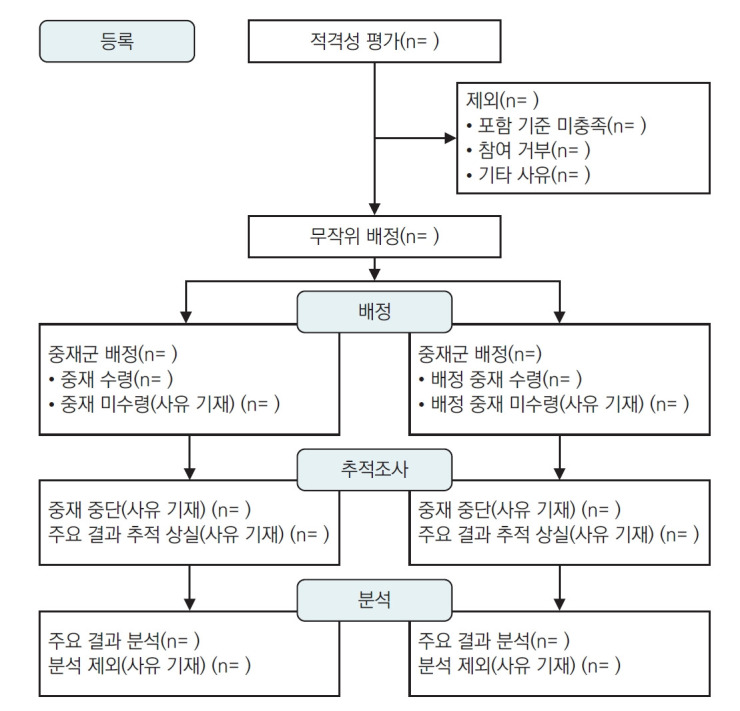
CONSORT 2025 흐름도. 두 그룹 무작위 배정 임상시험의 단계별 진행 상황(즉 등록, 중재 할당, 추적 관찰 및 데이터 분석)을 나타내는 흐름도. CONSORT, Consolidated Standards of Reporting Trials.

**표 1.** 무작위 임상시험을 보고할 때 포함해야 할 정보에 대한 CONSORT 2025 체크리스트

섹션/주제	No.	CONSORT 2025 체크리스트 항목 설명
제목 및 초록		
제목 및 구조화된 초록	1a	무작위 임상시험으로서의 식별
	1b	임상시험 설계, 방법, 결과 및 결론의 구조화된 요약
오픈 사이언스		
임상시험 등록	2	임상시험 등록부 이름, 식별번호(URL 포함) 및 등록날짜
프로토콜 및 통계 분석계획	3	시험 프로토콜 및 통계 분석계획에 접근할 수 있는 위치
데이터 공유	4	비식별화된 개별 참가자 데이터(데이터 사전 포함), 통계 코드 및 기타 자료에 접근할 수 있는 위치 및 방법
자금 및 이해관계	5a	자금 및 기타 지원 출처(예: 의약품 공급) 및 시험의 설계, 수행, 분석 및 보고에서 자금 제공자의 역할
	5b	원고 저자의 재정 및 기타 이해관계
서론		
배경 및 근거	6	과학적 배경 및 근거
목적	7	이익 및 위해와 관련된 구체적인 목표
방법		
환자 및 대중 참여	8	시험의 설계, 수행 및 보고에 대한 환자 또는 대중 참여 세부 사항
시험 설계	9	시험 유형을 포함한 시험 설계 설명(예: 평행군, 교차), 할당 비율 및 프레임워크(예: 우월성, 동등성, 비열등성, 탐색적)
시험 프로토콜 변경	10	시험 시작 후 미리 지정되지 않은 결과 또는 분석을 포함한, 시험에 대한 중요한 변경사항 및 이유
시험 설정	11	설정(예: 지역사회, 병원) 및 위치(예: 국가, 시험기관)
자격 기준	12a	참가자 자격 기준
	12b	해당되는 경우, 기관 및 중재를 제공하는 개인(예: 외과의, 물리치료사)에 대한 자격 기준
중재 및 비교군	13	복제를 허용하기에 충분한 세부 정보가 포함된 중재 및 비교군. 해당되는 경우, 중재 및 비교자를 설명하는 추가 자료(예: 중재 매뉴얼)에 접근할 수 있는 곳
결과	14	특정 측정 변수(예: 수축기 혈압), 분석 지표(예: 기준치, 최종값, 이벤트까지의 시간 변화), 집계방법(예: 중앙값, 비율) 및 각 결과에 대한 시점
위해	15	위해를 정의하고 평가한 방법(예: 체계적, 비체계적)
표본 크기	16a	표본 크기 계산을 뒷받침하는 모든 가정을 포함하여 표본 크기를 결정한 방법
	16b	중간 분석 및 중단 지침에 대한 설명
무작위 배정:		
시퀀스 생성	17a	무작위 할당 시퀀스를 생성한 사람 및 사용된 방법
	17b	무작위 할당 유형 및 제한 사항(예: 층화, 차단 및 블록 크기)
할당 은폐 메커니즘	18	무작위 할당 시퀀스를 구현하는 데 사용된 메커니즘(예: 중앙 컴퓨터/전화, 순차적으로 번호가 매겨진 불투명하고 밀폐된 용기), 중재가 배정될 때까지 순서를 은폐하는 모든 단계를 설명
구현	19	등록한 직원과 참여자를 중재에 배정하는 직원이 무작위 배정 순서에 접근할 수 있는지 여부
눈가림	20a	중재 배정 후의 눈가림 대상(예: 참가자, 의료 제공자, 결과 평가자, 데이터 분석가)
	20b	눈가림이 이루어진 경우, 눈가림이 어떻게 시행되었는지와 중재 간 유사성에 대한 설명
통계방법	21a	위해를 포함한 일차 및 이차 결과에 대한 그룹을 비교하는 데 사용한 통계적 방법
	21b	각 분석에 포함되는 사람(예: 모든 무작위 참가자) 및 그룹에 대한 정의
	21c	분석에서 누락된 데이터를 처리한 방법
	21d	추가 분석을 위한 방법(예: 하위 그룹 및 민감도 분석), 사전 지정과 사후 분석을 구분
결과		
흐름도를 포함한 참가자 흐름	22a	각 그룹에 대해 무작위로 배정되고, 의도된 중재를 받고, 일차 결과 분석에 포함된 참가자 수
	22b	각 그룹에 대해 무작위 배정 후의 손실 및 제외와 그 이유
모집	23a	혜택 및 피해 결과에 대한 모집 및 추적 기간을 정의하는 날짜
	23b	관련이 있는 경우 시험이 종료되거나 중단된 이유
중재 및 비교 대상 제공	24a	실제로 시행된 중재 및 비교 대상(예: 적절한 경우, 누가 중재/비교약을 제공했는지, 참가자가 어떻게 준수했는지, 의도대로 제공되었는지 여부[충실도])
	24b	각 그룹이 시험기간에 받은 병용 치료
기준 데이터	25	각 그룹의 기준 인구통계 및 임상 특성을 보여주는 표
분석된 숫자, 결과 및 추정	26	각 1차 및 2차 결과, 그룹별로:
		- 분석에 포함된 참가자 수
		- 결과 시점에 사용 가능한 데이터를 가진 참가자 수
		- 각 그룹에 대한 결과 및 추정 효과 크기와 그 사전값
위해	27	각 그룹에서 발생한 모든 위해 또는 의도치 않은 사건
보조 분석	28	사전 지정 분석과 사후 분석을 구별하여 수행된 기타 분석(하위 그룹 및 민감도 분석 포함)
고찰		
해석	29	결과에 부합하고, 이점과 위해의 균형을 맞추며, 기타 관련 근거를 고려한 해석
한계	30	잠재적 편향, 부정확성, 일반화 가능성 및 관련 있는 경우, 분석의 다중성 원인을 다루는 시험 한계

## Supplementary materials

Supplement 1. Comparison of CONSORT 2025 and CONSORT 2010 checklists.

Supplement 2. CONSORT 2025 expanded checklist of detailed information to include when reporting a randomised trial.
